# Utility of diffusion weighted imaging with the quantitative apparent diffusion coefficient in diagnosing residual or recurrent hepatocellular carcinoma after transarterial chemoembolization: a meta-analysis

**DOI:** 10.1186/s40644-019-0282-9

**Published:** 2020-01-06

**Authors:** Zhao Liu, Jin-Ming Fan, Chen He, Zhi-Fan Li, Yong-Sheng Xu, Zhao Li, Hai-Feng Liu, Jun-Qiang Lei

**Affiliations:** 1 The first Clinical Medical College of Lanzhou University, Lanzhou, 730000 Gansu China; 2 First Hospital of Lanzhou University, Lanzhou, 730000 Gansu China; 3 Department of Radiology, Third Affiliated Hospital of Soochow University & Changzhou First People’s Hospital, No.185, Juqian Street, Tianning District, Changzhou, 213003 Jiangsu China

**Keywords:** Diffusion weighted imaging (DWI), Apparent diffusion coefficient (ADC), Hepatocellular carcinoma (HCC), Transarterial chemoembolization (TACE), Meta-analysis

## Abstract

**Background:**

Accurate and early diagnosis of residual tumors or intrahepatic recurrences after TACE is critically needed for determining the success of treatments and for guiding subsequent therapeutic planning. This meta-analysis was performed to assess the efficacy of diffusion weighted imaging (DWI) with the quantitative apparent diffusion coefficient (ADC) value in diagnosing residual or recurrent hepatocellular carcinoma after transarterial chemoembolization (TACE).

**Materials and methods:**

A comprehensive literature search of PubMed, Embase, Web of Science, Scopus and the Cochrane Library database, from inception to July 2019, was conducted to select original studies on diagnosing residual or recurrent HCCs after TACE using DWI sequence with its ADC value. Two researchers independently chose study, extracted data, conducted meta-analysis, and evaluated methodological quality according to Quality Assessment of Diagnostic Accuracy Studies-2 (QUADAS-2) tool.

**Results:**

Twelve studies comprising 624 patients and 712 tumors were finally included. The pooled sensitivity, specificity and AUC value of DWI in diagnosing residual or recurrent HCCs after TACE were 85% (95%CI: 74–92%), 83% (95%CI: 75–88%) and 0.90 (95%CI: 0.87–0.92), respectively. Residual or recurrent HCCs have significantly lower ADC value than necrotic tumors (MD = -0.48, 95%CI: − 0.69~ − 0.27, *P* < 0.01).

**Conclusion:**

This study demonstrated that DWI performed better in diagnosing residual or recurrent HCCs after TACE, and ADC value may serve as alternatives for further evaluation of residual or recurrent leisions in HCC patients after TACE.

## Introduction

Hepatocellular carcinoma (HCC) is one of the most malignant tumors, with serious threats to human life and health, and it is estimated to be the fourth major factor of cancer death in the worldwide population [1]. However, only approximately 20% of patients with HCC are suitable for surgical resection or liver transplantation, mainly because the tumors are often diagnosed at an intermediate to advanced stage, and the associated liver cirrhosis is too far developed to permit tumor resection or liver transplantation to be endured [2, 3].

As a first-line and curative therapy, transarterial chemoembolization (TACE) operates by injecting chemotherapeutic drugs at the HCC site while impeding blood supply to the tumor. In previous studies, TACE has been proven to be an efficient bridge therapy for patients waiting for liver transplantation and can improve survival prognosis in patients with inoperable HCC [4, 5]. However, the rate of recurrent HCC after TACE is relatively high, with reported 12-month recurrence rates of 78% [6]. Therefore, the accurate and early diagnosis of residual tumors or intrahepatic recurrences after TACE is critically needed for determining the success of treatments and for guiding subsequent therapeutic planning.

By detecting water diffusion indirectly, diffusion weighted imaging (DWI) provides information regarding tissue cellularity and cell membrane integrity, and its quantitative value of the apparent diffusion coefficient (ADC) is highly associated with tumor cellularity [7, 8], indicating that DWI and ADC values may be potentially used to diagnose and differentiate residual or recurrent HCCs after TACE. Moreover, DWI and ADC values are of great importance in guiding the post-TACE treatment of patients with HCC. However, the accuracy of DWI and the quantitative ADC value for diagnosing residual or recurrent HCCs has shown conflicting results in an increasingly large number of clinical trials, mainly because different diagnostic accuracies associated with DWI and ADC values have been reported [9–20]. Therefore, based on the currently available published articles on DWI and the ADC value in detecting residual or recurrent HCCs after TACE, this meta-analysis was performed to provide evidence-based conclusions for imaging diagnostics.

## Materials and methods

### Literature search

According to the Preferred Reporting Items for Systematic reviews and Meta-Analyses (PRISMA) guidelines [21], a comprehensive literature search in PubMed, Embase, Web of Science, Scopus and the Cochrane library database was performed to select original studies, from inception to July 2019, that evaluated the accuracy of DWI and the quantitative ADC value in diagnosing residual or recurrent HCCs after TACE. Medical subject heading words and free words were used conjunctly and were as follows: (1) “Diffusion weighted imaging” or “DWI”; and (2) “transarterial chemoembolization” or “transcatheter arterial chemoembolization” or “TACE”. In addition, a manual search was performed of the identified review articles and included the study references to acquire available studies and to minimize the potential publication bias.

### Selection of articles

#### Inclusion criteria

(1) DWI and the calculated ADC values were used as diagnostic indices for detecting residual or recurrent HCCs after TACE. (2) Pathology and/or digital subtraction angiography (DSA) and/or follow-up clinical results served as the reference standard [22]. (3) The two-by-two table, including the true-positive (TP), false-positive (FP), false-negative (FN) and true-negative (TN) values, could be extracted or calculated. (4) More than 10 patients were included in the study. (5) When the data were published repeatedly, the latest study with detailed information was included. (6) The study was published in English.

#### Exclusion criteria

(1) Surgical resection and any other therapies besides TACE were applied to treat HCC. (2) The two-by-two table of DWI alone could not be acquired when DWI was used in conjunction with other MRI images; these studies were also excluded. (3) Review articles, case reports, animal studies, and editorial comments were excluded. (4) Conferences abstracts were also excluded, as their conclusions might not be up to date.

### Data extraction and quality assessment

Two researchers (ZL and HFL) independently extracted the study data and evaluated the methodological quality using the Quality Assessment of Diagnostic Accuracy Studies-2 (QUADAS-2) tool [23], and any disagreements were solved through discussion until consensus was reached. A predefined Excel file was adopted to extract study data, including first author name, publication year, country, study design type (prospective or retrospective), baseline information of involved patients (number, mean age, gender, the number of lesions), time interval between TACE and DWI examination, blinding method application, reference standard, DWI protocol (field strength, b value, ADC cut-off value), and threshold method used (visual diagnosis or ADC measurement). Additionally, two-by-two table values were extracted from all included studies. The quality was assessed via the evaluation of risk bias in four domains (patient selection, index test, reference standard, and flow and timing) and clinical applicability in three domains (patient selection, index test and reference standard) of the study characteristics. Each domain was scored as high, low, or unclear.

### Data synthesis and statistical analysis

Data synthesis and statistical analysis were performed using STATA (version 12.0, StataCorp, USA) and Review Manager software (version 5.3, the Cochrane Collaboration, 2014). To present the study characteristics of DWI in diagnosing residual or recurrent HCCs after TACE suitably, data synthesis was performed by the METANDI module and hierarchical logistic regression modeling in STATA software. The Spearman correlation coefficient was calculated to test the threshold, and a *P*-value less than 0.05 indicated the threshold effect. A chi-squared test was performed to measure the heterogeneity degree of the enrolled studies, and the degree was considered low for I-squared (*I*^2^) = 25 to 49%, moderate for *I*^2^ = 50 to 74%, and high for *I*^2^ > 75%.

The pooled sensitivity (SEN), specificity (SPE), positive likelihood ratio (PLR) and negative likelihood ratio (NLR) with their 95% confidence intervals (CIs) were computed from the two-by-two table data. Then, the receiver operator characteristics (ROC) curve and area under the ROC curve (AUC) were computed to evaluate the value of DWI in diagnosing residual or recurrent HCCs after TACE, and the value was considered good for AUC values > 0.9 and medium for AUC values from 0.7 to 0.9. Moreover, the ADC values of the residual or recurrent HCC group and the necrotic lesion group were compared and pooled as the mean difference (MD) with a random-effects model to incorporate expected heterogeneity. Additionally, possible factors, including study design type (retrospective or prospective), b value (< 800 or ≥ 800) and threshold method (visual analysis or ADC measurement), that may lead to heterogeneity were analyzed through subgroup analysis. Moreover, the reliability and stability of this study were evaluated through sensitivity analysis. Last, Deeks’ funnel plot with the linear regression of log odds ratios on the inverse root of effective sample sizes was performed to test for publication bias: a *P*-value < 0.05 was representative of publication bias.

## Result

### Study selection and description

**Fig. 1:**
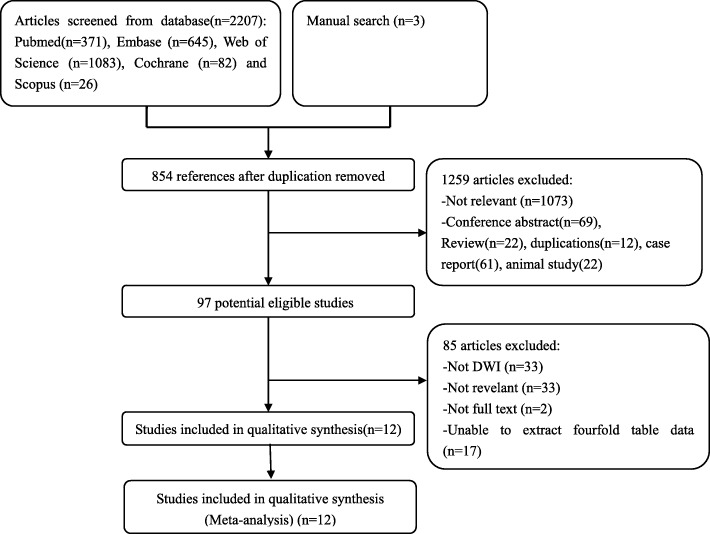
The flowchart of the study selection process

Figure 1 demonstrates a detailed flowchart of the study selection process. The electronic search combined with the manual search initially yielded 2210 potential literature references, and this number was reduced to 1356 after removing 854 duplicated references by Endnote X7 software. Upon reading the titles and abstracts, only 97 references were retrieved for further inspection. After full text review, a total of 12 studies [9–20], comprising 624 patients and 712 tumors evaluated by DWI, were finally included. The baseline information extracted from each study is presented in Table 1.

**Table. 1:** Baseine characteristics of included studes

Author	Year	Country	Design	No. Patient	Gender	Age, Year	time interval	No. lesion	Field Strength	b value	Blind	Reference Standard	ADC cut off value (×10^−3^ mm^2^/s)	Threshold used
Afifi [9]	2016	Egypt	Pro	20	18/2	56(42–69)	3w	20	1.5 T	0, 300,600	NR	follow up	NR	Visual
Du [10]	2014	China	Re	89	57/32	56.0(26–78)	1mo	113	1.5 T	0,800	NR	follow up	1.54	ADC
Ebraheem [11]	2017	Egypt	Pro	50	42/8	60(40–79)	NR	50	1.5 T	0,50,1000	NR	follow up	NR	Visual
Goshima [12]	2008	Japan	Re	25	18/7	60(48–79)	2-6mo	39	1.5 T	0,500	Y	follow up	NR	Visual
Hassan [13]	2019	USA	Re	60	45/15	60(40–73)	NR	63	1.5 T	0,400,800	NR	follow up	N	Visual
Kokabi [14]	2015	Georgia	Pro	57	39/18	61.3(25–82)	3mo	57	1.5 T	50,400,800	NR	follow up	0.83	ADC
Li [15]	2016	China	Pro	117	86/31	51.7(31–74)	1mo	117	NR	NR	NR	follow up	1.24	ADC
Mannelli [16]	2009	USA	Re	21	19/2	57.7(30–70)	10d-3mo	28	1.5 T	0,50,500	Y	Patholoy	2.16	ADC
Wu [17]	2017	China	Pro	84	57/27	54.5(25–76)	1mo-2mo	84	1.5 T	300,600,800	NR	follow up	1.20	ADC
Xiao [18]	2008	China	Re	15	13/2	45.7(17–63)	10d-2mo	30	1.5 T	0,500	Y	patholoy	NR	Visual
Yousef [19]	2017	Egypt	Pro	45	38/7	59(38–75)	3w-5 m	59	1.5 T	0,400,800	NR	follow up	NR	Visual
Yuan [20]	2014	China	Pro	41	34/7	56.2(23–78)	6w-8w	52	1.5 T	0,500	Y	follow up	1.84	Visual

*No* Number, *Pro* Prospective, *Re* Retrospective, *NR* Not report, *ADC* Apparent diffusion coefficient

### Assessment of study quality

The methodological assessment results of the included studies are shown in Table 2. The risk of bias for patient selection was primarily related to case-control studies. Case-control studies were defined as studies involving both patients prediagnosed with and patients without residual or recurrent HCCs after TACE before undergoing DWI examination. In this meta-analysis, none of the included studies had a case-control design, leading to a low risk of bias in patient selection for all included studies. For this meta-analysis, only four studies showed a clear blinding application method [12, 16, 18, 20], which results in an unclear risk of bias concerning the index test in the other eight studies. Pathology, DSA and clinical follow-up results are all reliable reference standards in diagnosing residual or recurrent HCCs after TACE; therefore, all studies included in this meta-analysis presented a low risk of bias compared with the reference standard.

**Table. 2:** The distribution of included quality according to QUADAS-2 tool

	Afifi 2016 [9]	Du 2014 [10]	Ebraheem 2017 [11]	Goshima 2008 [12]	Hassan 2009 [13]	Kokabi 2015 [14]	Li 2016 [15]	Mannelli 2009 [16]	Wu 2017 [17]	Xiao 2008 [18]	Yousef 2017 [19]	Yuan 2014 [20]
Ris of bias	U	U	U	L	U	U	U	L	U	L	U	L
Patient selection	L	L	L	L	L	L	L	L	L	L	L	L
Index Test	U	U	U	L	U	U	U	L	U	L	U	L
Reference standard	L	L	L	L	L	L	L	L	L	L	L	L
Flow and timing	L	L	L	L	L	L	L	L	L	L	L	L
Applicability Concerns	L	L	L	L	L	L	L	L	L	L	L	L
Patient selection	L	L	L	L	L	L	L	L	L	L	L	L
Index Test	U	U	U	L	U	U	U	L	U	L	U	L
Reference standard	L	L	L	L	L	L	L	L	L	L	L	L

*L* Low, *U* Unclear

### Statistical analysis

#### Heterogeneity test

The Spearman correlation coefficient was 0.141 (*P* = 0.662 > 0.05), indicating that there was no obvious threshold effect of DWI in diagnosing residual or recurrent HCCs after TACE. The chi-squared value of the pooled SEN was 27.47 (*P* = 0.02 < 0.05), and the *I*^2^ value was 49.03%, demonstrating low heterogeneity for SEN. SPE had a chi-squared value of 56.58 (*P* < 0.01), and the *I*^2^ value was 75.38%, representing high heterogeneity.

#### Pooled analysis

**Fig. 2:**
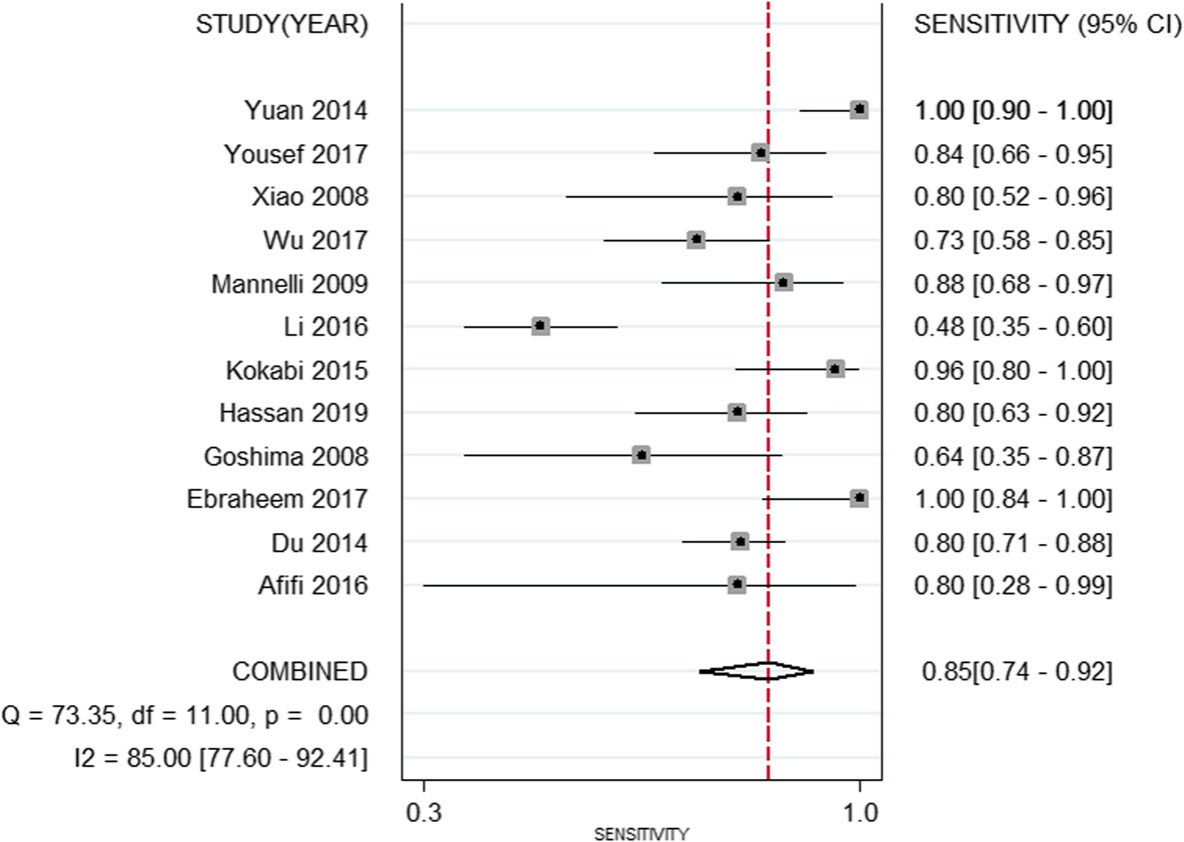
Forest plots of sensitivity for DWI in diagnosing residual or recurrent HCCs after TACE

**Fig. 3:**
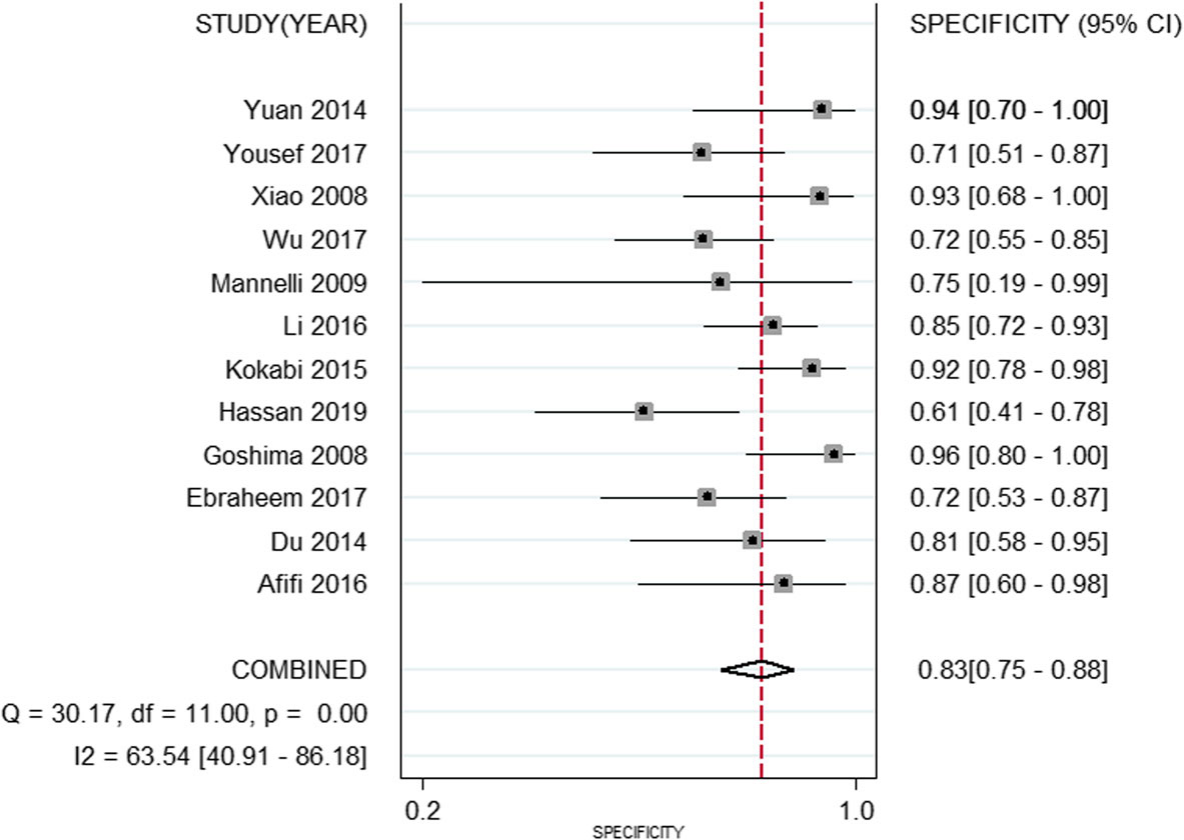
Forest plots of specificity for DWI in the diagnosis of residual or recurrent HCCs after TACE

**Fig. 4:**
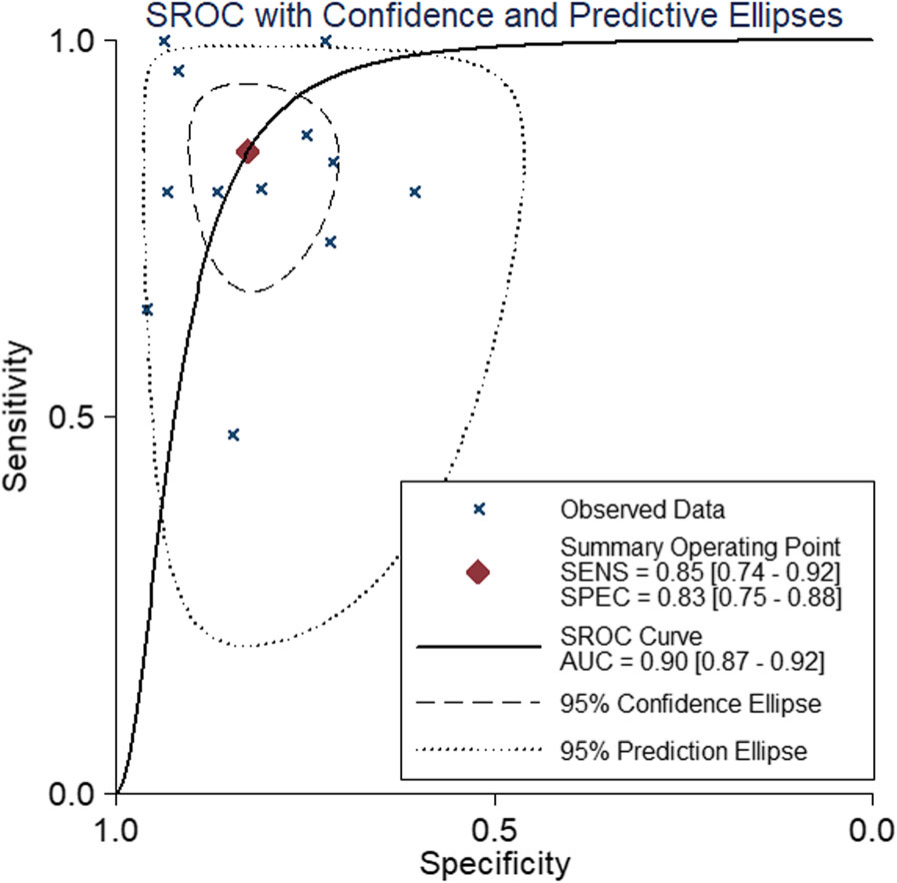
Forest plots of ROC for DWI in the detection of residual or recurrent HCCs after TACE

The pooled weighted values of DWI in diagnosing residual or recurrent HCCs after TACE were as follows: SEN =85% (95% CI: 74–92%), SPE =83% (95% CI: 75–88%), PLR =5.12 (95% CI: 3.27–7.38), NLR =0.18 (95% CI: 0.09–0.34), and AUC =0.90 (95% CI: 0.87–0.92). Forest plots and ROC curves for the 12 studies are shown in Figs. 2, 3, 4.

### Comparison of ADC values

**Fig. 5:**
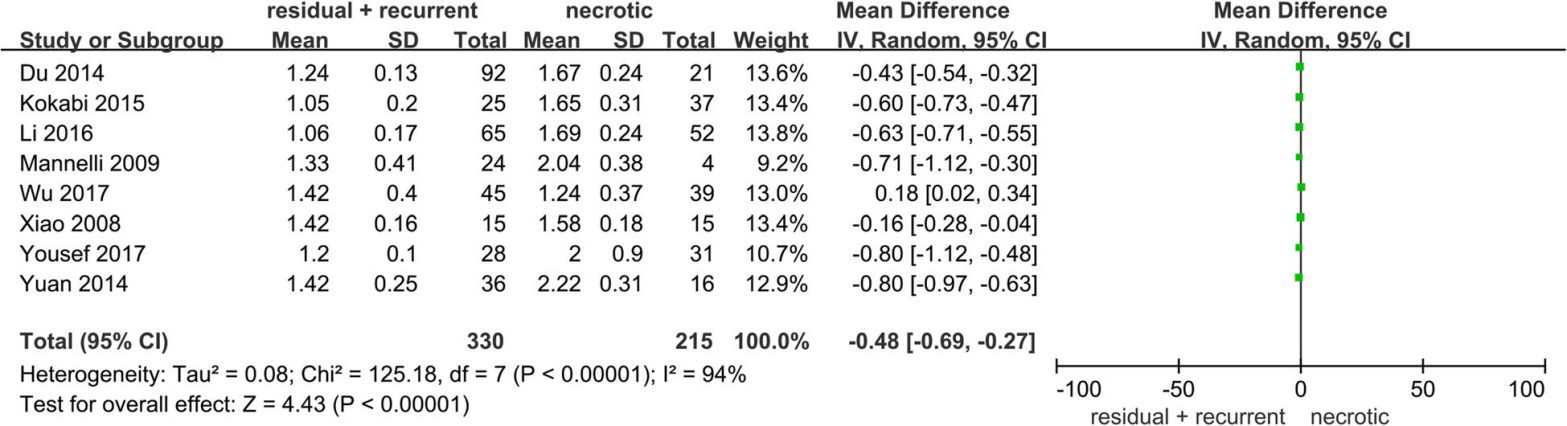
Forest plots of ADC value between residual or recurrent HCCs and necrotic tumors

Among the included studies, 8 studies compared the ADC value between residual or recurrent HCCs and necrotic tumors. Our pooled analysis demonstrated that residual or recurrent HCCs had significantly lower ADC values than necrotic tumors (MD = -0.48, 95% CI: − 0.69~ − 0.27, *P* < 0.01), which is shown in Fig. 5, indicating that the ADC value may be important for differentiating residual and recurrent HCCs after TACE.

### Subgroup analysis

As presented in Table 3, the study design type, b value and threshold method used did not significantly affect the ability of DWI to diagnose residual or recurrent HCCs after TACE.

**Table 3:** Subgroup analysis for the diagnostic performance of DWI on overall level

Characteristic	No.of Studies	Sensitivity (95% CI)	Specificity (95% CI)	*I*^2^ index of Sensitivity/Specificity	AUC (95% CI)	*P* Value
All studies	12	0.85(0.74–0.92)	0.83(0.75–0.88)	85.0/65.3	0.90(0.87–0.92)	
Study design						
Retrospective	5	0.80(0.73–0.86)	0.81(0.71–0.88)	0.00/69.6	0.86(0.83–0.89)	0.68
Prospective	7	0.91(0.69–0.98)	0.82(0.74–0.88)	92.1/67.5	0.88(0.85–0.90)	
b value						
< 800	5	0.88(0.69–0.96)	0.92(0.83–0.96)	67.5/0.00	0.93(0.91–0.95)	0.40
≥ 800	6	0.85(0.75–0.92)	0.76(0.66–0.83)	58.8/49.2	0.87(0.83–0.89)	
Threshold method						
visual	6	0.82(0.71–0.89)	0.82(0.67–0.91)	38.663.1	0.88(0.85–0.90)	0.47
ADC	6	0.87(0.65–0.96)	0.85(0.76–0.91)	92.9/72.7	0.90(0.87–0.92)	

*P* value: Comparasion of AUC value between different subgroup analyses

### Analysis of sensitivity and publication bias

**Fig. 6:**
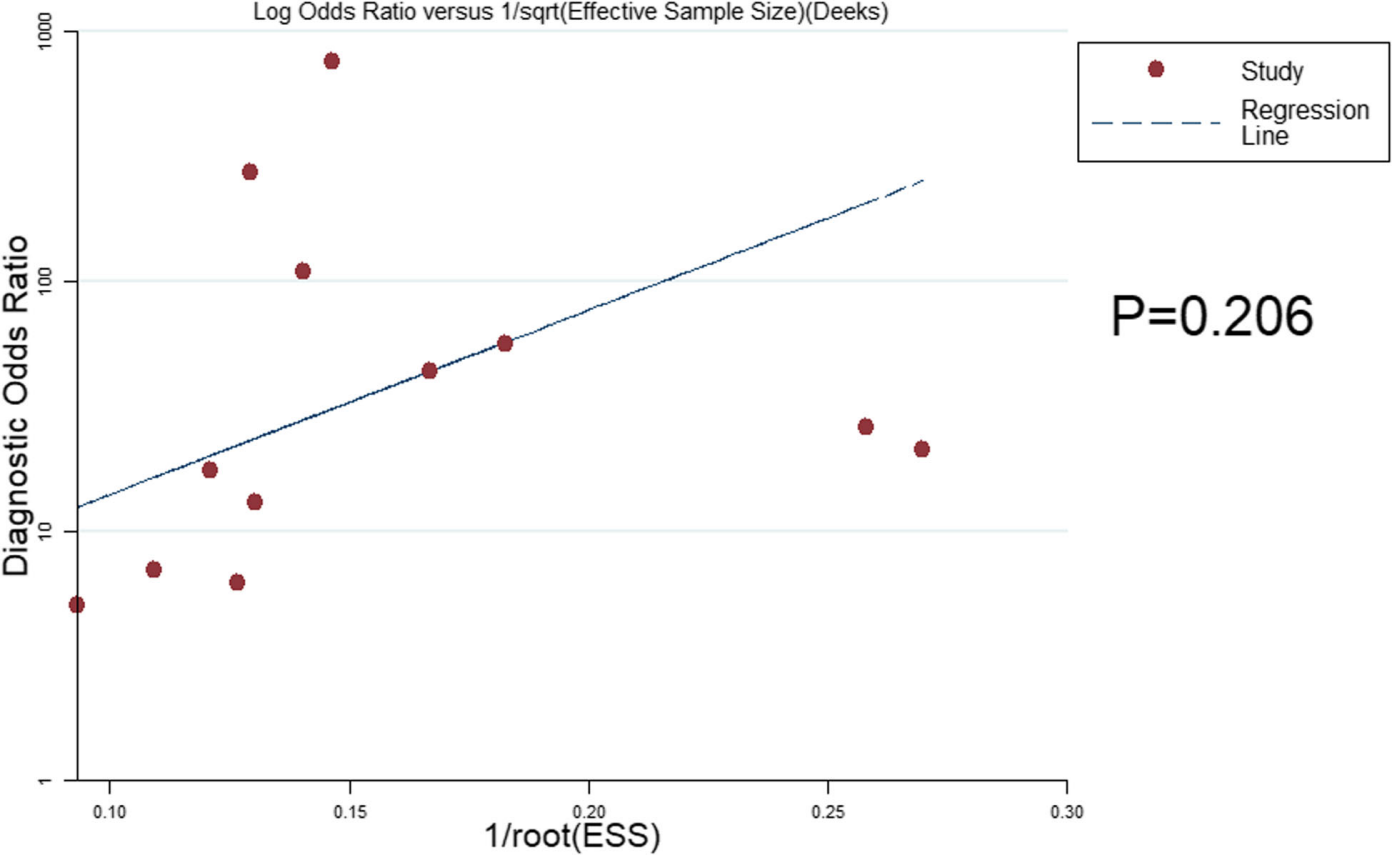
Deeks’ funnel plot for publication bias assessment of DWI for diagnosis of residual or recurrent HCCs after TACE

For the use of DWI in the detection of residual or recurrent HCCs after TACE, the reliability and stability of this meta-analysis were assessed by removing one study with unclear field strength and the use of the b value. The resulting SEN was 87% (95% CI 78–93%), SPE was 83% (95% CI 74–89%) and AUC was 0.91 (95% CI 0.88–0.93), indicating that the effect values were still within the 95% CI of the original results, thus showing that the results were reliable and stable. Deeks’ funnel plot is presented in Fig. 6 and demonstrates no obvious publication bias of DWI in the diagnosis of residual or recurrent HCCs after TACE (*P* = 0.206).

## Discussion

Because of the potential severe complications and relatively high cost, pathology and digital subtraction angiography are not recommended as routine follow-up examinations for diagnosing residual or recurrent HCCs after TACE [24]. However, residual or recurrent HCCs increase the disease burden and worsen the survival prognosis of patients after TACE [25]; thus, noninvasive imaging is increasingly being used to diagnose residual or recurrent HCCs after TACE. Contrast-enhanced computed tomography (CECT) is commonly used in analyzing the distribution of lipiodol deposition and is beneficial for evaluating the therapeutic effects of TACE [26]. However, lipiodol deposition often makes it hard to diagnose the residual tumor on CECT because the accumulation of intratumoral lipiodol may mask its enhancement, which would significantly decrease the accuracy and contribute to a lower sensitivity of 72% (95% CI: 67–76%) [27] compared with this study conclusion that DWI had a higher sensitivity of 85% (95% CI: 74–92%) in detecting residual or recurrent HCCs. Contrast-enhanced ultrasonography (CEUS) is a valuable imaging method for evaluating vascularity because the depiction of tumor vascularity is not affected by lipiodol accumulation. A recent meta-analysis reported that CEUS can reach up to a weighted SEN of 97% (95% CI: 95–99%) in the diagnosis of residual or recurrent HCC after TACE [27]. Nevertheless, CEUS examination has always been conducted on a solitary mass or on a dominant mass in patients with multiple tumors, which limits its wide clinical application in detecting residual or recurrent HCCs after TACE [28].

After the TACE procedure, the HCC tumor cells undergo necrosis and apoptosis, with increased cell membrane permeability and enlarged cell gaps, leading to more water molecule movement on DWI and an increased ADC value. For residual or recurrent HCCs, the tortuosity of the extracellular space and the higher density of hydrophobic cellular membranes will increase cell density and restrict the apparent diffusion of water protons, thus presenting a high signal on DWI and a lower ADC value [7, 29]; therefore, DWI was increasingly used in the follow-up of TACE treatment. To provide a diagnostic value and to investigate the possible factors that affect the efficacy of conducting DWI, this meta-analysis was performed to evaluate the value of DWI in diagnosing residual or recurrent HCCs after TACE.

In this study, 12 studies comprising 624 patients and 712 tumors detected on DWI were included. The SEN, SPE, and AUC values of DWI in diagnosing residual or recurrent HCCs after TACE were 85% (95% CI: 74–92%), 83% (95% CI: 75–88%), and 0.90 (95% CI: 0.87–0.92), respectively, indicating a high value of DWI in diagnosing residual or recurrent HCCs after TACE. Additionally, the PLR for DWI was 5.12 (95% CI: 3.27–7.38), revealing moderate accuracy in diagnosing residual or recurrent HCCs after TACE. The NLR value for DWI was 0.18 (95% CI: 0.09–0.34), and a negative DWI result may be used as a moderate justification to rule out residual or recurrent HCCs after TACE. Moreover, this pooled analysis also indicated that the ADC value of necrotic tumors was significantly higher than that of residual or recurrent HCCs, confirming previous findings and revealing that the ADC value can be used as an efficient imaging method to differentiate the properties of lesions after TACE.

In this meta-analysis, the opposite results of false-negative (1-SEN) and false-positive (1-SPE) rates were 15 and 17%, respectively, for DWI in diagnosing residual or recurrent HCCs after TACE, and some of the following possible factors could contribute to the relatively high number of false-negative and false-positive results. 1) Adjacent hepatic inflammation could restrict water diffusion, resulting in sustained hyperintensity on DWI, thereby producing a false-positive DWI diagnosis of the lesion after TACE [30, 31]. 2) Intralesional hemorrhage or liquefactive necrosis can occur after TACE, which may also contribute to diffusion restriction in necrotic tumors, and can decrease the accuracy of DWI in the detection of residual or recurrent HCCs [32]. 3) Owing to the limited spatial resolution of DWI, small tumors would not be diagnosed precisely, leading to false-negative and false-positive results on DWI [33]. 4) The signal intensities observed on DWI are easily affected by the T2-relaxation time of the tissue, as seen in the fact that a false-positive diagnosis of necrotic tissue may be induced by well-differentiated HCCs, high-grade dysplastic nodules, and hemangioma [34]. Only histopathology can be regarded as the absolute gold standard for diagnosing residual or recurrent HCCs [22]. DSA or follow-up results may contribute to false-positive results.

Significant sources of heterogeneity concerning the use of DWI in the diagnosis of residual or recurrent HCCs after TACE and the evaluation of this meta-analysis demonstrated 4 potential factors that may account for this result: 1) Freiman et al. [35] reported that a higher b value might decrease the signal-to-noise ratio (SNR), whereas blood perfusion can be easily affected by a lower b value. Vandecaveye et al. [36] suggested that the SNR is the highest and the image quality of DWI is the best at a specific b value of 600 s/mm^2^. This meta-analysis demonstrated that DWI performed better for the diagnosis of residual or recurrent HCCs after TACE with lower b values than with higher b values, revealing that the b value of DWI was one of the factors affecting diagnostic efficacy. 2) Heterogeneity might be induced by the different threshold methods used. There is a trend towards a higher diagnostic value of ADC measurement compared to visual diagnosis. Visual diagnosis is easily affected by the T2 shine-through effect and may account for this result [37]. 3) There are 5 retrospective studies and 7 prospective studies included in this study, which may be another reason for the heterogeneity. Prospective studies tended to perform better, although these findings were not statistically significant. 4) Heterogeneity might also be induced by the various field strengths adopted in the different studies [38], because a higher resolution of images will be created with higher field DWI-MRI scanners. However, we were not able to perform subgroup analysis associated with field strength owing to the limited included studies.

There were some deficiencies in this study that should be noted. First, although a combination of electronic and manual searches was performed, only 12 studies comprising 624 patients on DWI were involved. The small number of included studies might have a negative impact on the diagnostic accuracy; therefore, further high-quality studies on a larger scale may be required. Second, the exclusion of studies other than those published in English, review articles and conference abstracts may contribute to potential publication bias. Third, field strength was found to affect the efficacy of DWI in diagnosing residual or recurrent HCCs after TACE. However, because of the limitations of the included studies, the accuracy of DWI in diagnosing residual or recurrent HCCs after TACE from the perspective of field strength was not been explored.

## Conclusion

In conclusion, our study suggested that DWI performs well in diagnosing residual or recurrent HCCs after TACE, and the ADC value may serve as an alternative marker in the further evaluation of HCC patients after TACE.

## Data Availability

The datasets used and/or analyzed during the current study are available from the corresponding author on reasonable request.

## Abbreviation

ADC: Apparent diffusion coefficient
AUC: Area under the curve
CI: Confidence interval
DSA: Digital subtraction angiography
DWI: Diffusion weighted imaging
FN: False negative
FP: False positive
HCC: Hepatocellular carcinoma
MD: Mean difference
ROC: Receiver operator characteristics
TACE: Transarterial chemoembolization
TN: True negative
TP: True positive
